# Cognitive decline in Parkinson’s disease is associated with reduced complexity of EEG at baseline

**DOI:** 10.1093/braincomms/fcaa207

**Published:** 2020-11-27

**Authors:** Sebastian M Keller, Ute Gschwandtner, Antonia Meyer, Menorca Chaturvedi, Volker Roth, Peter Fuhr

**Affiliations:** 1 Department of Mathematics and Computer Science, University of Basel, Basel 4031, Switzerland; 2 Department of Neurology, University Hospital Basel, Basel 4031, Switzerland

**Keywords:** EEG, Parkinson’s disease, cognitive decline, signal complexity, Tsallis entropy

## Abstract

Parkinson’s disease is a neurodegenerative disorder requiring motor signs for diagnosis, but showing more widespread pathological alterations from its beginning. Compared to age-matched healthy individuals, patients with Parkinson’s disease bear a 6-fold lifetime risk of dementia. For individualized counselling and treatment, prognostic biomarkers for assessing future cognitive deterioration in early stages of Parkinson’s disease are needed. In a case–control study, 42 cognitively normal patients with Parkinson’s disease were compared with 24 healthy control participants matched for age, sex and education. Tsallis entropy and band power of the δ, θ, α, β and γ-band were evaluated in baseline EEG at eyes-open and eyes-closed condition. As the θ-band showed the most pronounced differences between Parkinson’s disease and healthy control groups, further analysis focussed on this band. Tsallis entropy was then compared across groups with 16 psychological test scores at baseline and follow-ups at 6 months and 3 years. In group comparison, patients with Parkinson’s disease showed lower Tsallis entropy than healthy control participants. Cognitive deterioration at 3 years was correlated with Tsallis entropy in the eyes-open condition (*P* < 0.00079), whereas correlation at 6 months was not yet significant. Tsallis entropy measured in the eyes-closed condition did not correlate with cognitive outcome. In conclusion, the lower the EEG entropy levels at baseline in the eyes-open condition, the higher the probability of cognitive decline over 3 years. This makes Tsallis entropy a candidate prognostic biomarker for dementia in Parkinson’s disease. The ability of the cortex to execute complex functions underlies cognitive health, whereas cognitive decline might clinically appear when compensatory capacity is exhausted.

## Graphical Abstract

**Graphical Abstract:**
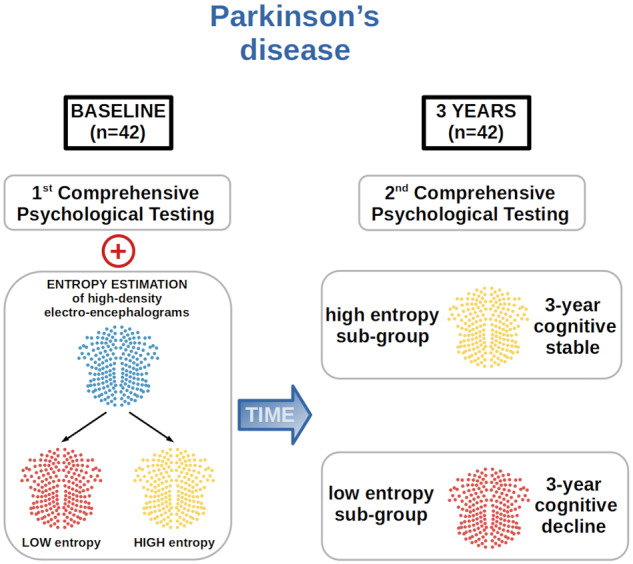

## Teaser

EEG-based Tsallis entropy measures signal complexity of brain rhythms. Low complexity of baseline EEG, measured in eyes-open condition, correlates with cognitive decline after 3 years in patients with Parkinson’s disease. Tsallis entropy of EEG is, therefore, a candidate prognostic biomarker for cognition in Parkinson’s disease.

## Introduction

While Alzheimer’s disease is the most common neurodegenerative disorder, Parkinson’s disease is the fastest growing one (Dorsey and Bloem, 2018). According to conservative estimates based on worldwide prevalence data from a 2014 meta-analysis (Pringsheim *et al.*, 2014), the number of people suffering from Parkinson’s disease is expected to reach 14.2 million in 2040, which would effectively double the number of cases compared to 2015. Despite being considered primarily a motor disorder, ∼30% of patients with Parkinson’s disease have cognitive symptoms already at initial diagnosis, and up to 80% develop cognitive symptoms at some point in their disease (Emre *et al.*, 2007; Hely *et al.*, 2008). The prognosis for losing independence or life currently depends much more on neuropsychiatric and cognitive deterioration than on motor signs (Forsaa, 2010; Bäckström, 2018). Moreover, cognition is an important aspect of quality of life for patients as well as their caregivers (Lawson *et al.*, 2016, 2017). Therefore, preservation and improvement of cognition in Parkinson’s disease patients have recently become major goals for therapeutic interventions and trials. Patient care and clinical trials regarding cognition currently rely mainly on bedside assessments and psychological testing with its known difficulties, including availability, reliability and test–retest biases. In contrast, biomarkers are objective monitors or predictors of the disease course and will improve making decisions for individual patients as well as for defining optimal populations for clinical trials for cognitive decline in Parkinson’s disease (Cramer, 2010; Dodakian *et al.*, 2013).

According to Stam (2014), normal cognition is characterized by electrical brain activity with an optimal degree diversity, order and hierarchy. Analogously, normal consciousness is characterized by an optimal entropy level of the EEG, whereas its reduction leads eventually to loss of consciousness and abnormal increase in incoherent thinking, such as in a psychedelic state of consciousness (Carhart-Harris *et al.*, 2014). Tsallis entropy (TE) of EEG, when measured during a recall task and characterized as a ratio between frontal and parietal regions, resulted in a very high accuracy for detection and treatment monitoring of mild cognitive impairment due to beginning stage of Alzheimer’s disease (Sneddon, 2005, 2007).

We therefore hypothesized *a priori* that TE of the EEG at baseline correlates with cognitive deterioration over a period of 3 years. Moreover, we hypothesized *a posteriori* that the decrease of entropy is a diffuse effect not attributable to a single location and that TE of the θ-band, measured during eyes-open (EO) condition, is significantly more informative about future cognitive performance than the same band evaluated in eyes-closed (EC) condition. Furthermore, we hypothesized that band power (BP), as a predictor for cognitive decline, is agnostic to the recording condition of EO versus EC.

## Materials and methods

### Participant demographic

The study is based on a cohort of 42 patients with Parkinson’s disease who were recruited from the Movement Disorders Clinic of University Hospital of Basel from 2011 to 2016 by advertising in the magazine of the Swiss Parkinson’s Disease Association. Parkinson’s disease was diagnosed according to the United Kingdom Parkinson’s Disease Brain Bank criteria (Gibb and Lees, 1988). Neuropsychological assessment was carried out in all individuals when they were admitted into the study (baseline), then at 6-month and at 3-year follow-ups. Knowledge of the German language was a prerequisite for the inclusion into this study. Patients with psychiatric or organic brain disease as well as patients with complete missing data at years follow-up were excluded from the analysis. The complete consort schema is shown in Supplementary Figure 5.

A group of 24 healthy controls (HC) matched for age, sex and education was recruited from the Memory Clinic, University of Basel Center for Medicine and Aging, and from the University Hospital of Basel. The demographic characteristics of the participants are summarized in Table 1. The studies were approved by the local ethics committee (Ethikkommission beider Basel, ref. no: 135/11, 294/13, 260/09). All participants gave their written informed consent.

**Table 1:** Values are presented as median values, along with values for the 25% and 75% quartiles

	Age	Education	Sex	Mini-mental state examination	UPRDS-III	LED	Disease duration	Sleepiness
HC (24)	66.5	14	9f.	30				
1st quartile	64	12		29				
3rd quartile	68.5	17.25		30				
Parkinson’s disease (42)	66.5	14	18f.	29	14.5	543	2.5	3
1st quartile	63	12		28	5	305	1	2.875
3rd quartile	72.75	16		30	21	1014	5	3.25

Age, education and disease duration are given in years. LED is given in milligrams. MMS and UPDRS-III refer to standardized psychological tests. Sleepiness is rated according to the Karolinska Sleepiness Scale (1—extremely alert; 10—extremely sleepy).

As all patients underwent comprehensive neuropsychological examinations, analyses showed that patients who performed all tests scored significantly higher in the Mini-Mental State Examination (median score = 30 versus 28; *W* = 319; *P* < 0.05), and had a lower disease duration (median score = 2 versus 4.5; *W* = 141.5; *P* < 0.05) than patients with incomplete data. No other differences in demographic or disease characteristics were observed between the two groups.

### Clinical, neurological and neuropsychological assessments

A basic neurological examination was carried out in all individuals. All patients underwent comprehensive neuropsychological examinations. The following cognitive domains were of interest for this study:

- *Attention and psychomotor speed:* Alertness (reaction time with and without sound) and Divided Attention (reaction time to visual and auditive stimulus, number of omissions) of the computerized ‘Test Battery of Attentional Performance’ (Zimmermann and Fimm, 2007), the Trail Making Test Part A (Reitan, 1958)
- *Executive functions and working memory:* phonemic (s-words, Thurstone and Thurstone, 1948) and semantic fluency (animals, Isaacs and Kennie, 1973), TAP Working memory (number of omissions), Digit span and Corsi Block (forward and backward) (Härting *et al.*, 2000)
- *Visuo-constructive abilities:* Block Design Test (Tewes, 1991)

A reliable change index (RCI) for each neuropsychological test was calculated for both the 6-month and 3-year follow-up after baseline. The individual RCI values were then combined into an overall RCI for the 6-month and 3-year follow-up. As the RCI is a standardized measure, combining multiple RCI values is done through simple averaging. The overall RCI was used as the outcome variable. The RCI for a single psychological test was calculated as the difference between the test score at either 6-month or 3-year follow-up and the test score at baseline, divided by the standard error of the difference (Jacobson and Truax, 1991):

$$SE_{M}=\mathrm{std}\left(\mathrm{baseline}\right)\times \sqrt{1-\mathrm{RS}}\;$$

$$S_{\mathrm{diff}}=\sqrt{2\times {\mathrm{SE}}_{M}^{2}}$$

$$\mathrm{RCI}\;=\;\frac{\mathrm{follow}-\mathrm{up}\;-\;\mathrm{baseline}}{S_{\mathrm{diff}}}$$

Equation 1: SE_M_: standard error of measurement; std(baseline): standard deviation of baseline; *S*_diff_: standard deviation of the errors of measurements; RS: reliability of measurement.

As 5.5% of the neuropsychological data points were missing, candidate values were generated based on the multiple imputation method for both predictors and missing outcomes (Little, 1992).

As a consequence, sample size was maintained and introducing potential biases, due to systematically missing data, was avoided.

### EEG recording and signal processing

A total of 20 min of EEG was recorded at wakeful rest for each patient by using a 256-channel EEG System (Netstation 300, EGI, Inc., Eugene, OR, USA). The recordings of EEG were done in the afternoons and patients were seated comfortably in a relaxing chair, instructed to open and close their eyes at regular intervals in the beginning, then closing their eyes for 15 min and opening them again towards the end (5 min). A technician present in the recording room controlled for vigilance of the patients and kept them alert. Before the EEG recording, patients were also asked to self-rate their sleepiness level from 1 to 10 by using the Karolinska Sleepiness Scale (Åkerstedt and Gillberg, 1990; Kaida *et al.*, 2006; Miley *et al.*, 2016).

All data were first separated into segments containing only recordings in either EO or EC condition. Subsequently, these segments were processed in an automated way by using the MATLAB based in-house software toolbox ‘TAPEEG’ (Hatz *et al.*, 2015), available at https://sites.google.com/site/tapeeg/. The recordings of EEG were filtered (Firls: 0.5–70 Hz, 50 Hz notch) at a sampling rate of 1000 Hz and an inverse Hanning window was used to stitch together shorter segments to have at least 3 min of cleaned EEG data. The implementation of independent component analysis (‘runica’) used for preprocessing was originally part of the toolbox ‘EEGLAB’ (Delorme and Makeig, 2004). ‘TAPEEG’, which combines methods from ‘EEGLAB’, ‘FASTER’ and ‘Fieldtrip’, was used for the entire preprocessing of EEG. As ‘TAPEEG’ is freely available (including handbook/tutorial), the full pre-processing pipeline can easily be reproduced. ‘TAPEEG’, with default settings, was used in order to detect bad channels/activations/segments. Furthermore, eye movement artefacts, traces of sleep, eye blinking, ECG and muscle artefacts were detected and removed. The average of all ‘good’ channels was used to re-reference the EEG to a common average montage. Electrodes placed on the neck, ears and cheeks were excluded to remove spurious signals, and 213 electrodes were mapped to 10 regions of interest: frontal left/right, central left/right, parietal left/right, temporal left/right and occipital left/right. For analysis, a total of 3 min of EEG in EC as well as EO condition was available per patient. In 3 of 66 cases, <180 s, but more than 170 s of EEG data were available, which did not affect the estimates of TE. The 66 artefact-free segments were then filtered into the following five bands: 1–4 Hz (δ-band), 4–8 Hz (θ-band), 8–13 Hz (α-band), 13–30 Hz (β-band) and 30–45 Hz (γ-band). For filtering, a zero-phase band pass FIR filter with Hann window was used.

### Statistical analysis

Calculation of TE was performed as described by Sneddon (2007). The algorithm was implemented in the Python (v3.5). Details are available in the supplementary material. R statistical software was used for analysis. For missing entries in test psychological data, the multiple imputation method implemented in the ‘MICE’ R-package (van Buuren and Groothuis‐Oudshoorn, 2011) was used to generate a total of 20 imputed data sets. Imputation of missing psychological test values is based on all available tests from baseline, 6-month and 3-year follow-ups. Linear regression analyses were performed on each imputed data set and pooled, again using the aforementioned R-package. Given the relatively small number of patients available, the number of potential confounders was reduced by adopting a strategy proposed by van Buuren (2018), consisting of a stepwise forward selection, performed on each imputed data set, in order to identify significant confounders. This was followed by a majority vote over all 20 data sets in order to identify significant confounders which appeared *consistently*, i.e. in the majority of imputed data sets. Finally, only confounders which appeared consistently were considered in the subsequent data analysis. Based on the ‘relaimpo’ R-package (Grömping, 2006), the relative importance of TE and BP as predictors as well as the importance of the confounders was assessed. Alpha, the probability of a Type I error, was 0.05. In case of multiple testing, the significance threshold was adjusted according to Holm–Sidak. Two-tailed hypothesis tests were considered throughout. If the requirements for the Welch *t*-test were not fulfilled, the non-parametric Mann–Whitney *U*-test was used. For the extreme groups, significances were not calculated. Following Preacher *et al.*’s (2005) descriptions, excluding already available data from the analysis by applying an artificial threshold would have resulted in inflated *P*-values.

### Data availability

Data supporting the findings of this study are available from the corresponding author, upon reasonable request.

## Results

### Tsallis entropy and relative band power provided non-redundant information

EEG spectral BP, which reflects the number of neurons that discharge synchronously, is by far the most commonly known feature in quantitative EEG analyses (Klimesch, 1999; Buzsáki and Draguhn, 2004). Based on the Berger effect (Berger, 1929), which describes a significant decrease in power of α-band oscillations when cognitively normal participants open their eyes, it can be shown that TE contains non-redundant information compared to BP. For the HC group of 24 participants, TE as well as relative BP were calculated in both EC and EO condition for each of the 213 electrodes in the δ, θ, α, β and γ-bands. Subsequently, the 213 TE and BP values were grouped into 10 non-overlapping regions (frontal, temporal, parietal, central and occipital, each left and right). For each participant, the average within each region was calculated separately for the EC and the EO condition. Then the differences were computed between the region-wise averages of both states, i.e. EO − EC. For each region, the mean response of the cohort, when transitioning from the EC into the EO condition, is obtained by averaging over the individual mean TE, respectively, BP values. Quantitatively, it is observed that the magnitude of change relative to the EC condition is completely different for TE compared to BP (P< 1e − 60). Most notably, while the relative BP of the θ-band is unaffected by the transitioning from EC into EO condition, TE of that same band increases considerably, especially in the frontal region. On the other hand, relative BP of the α-band strongly decreases during EO compared to EC condition, but TE increases. In summary, these observations showed that TE, in the frequency range from 1 to 13 Hz, is generally higher in EO condition, whereas for BP the same does not hold true. A visual comparison, including of all five bands, is provided in Supplementary Fig. 2.

### Tsallis entropy characterized the Parkinson’s disease and healthy control group in the θ-band

**Figure 1:**
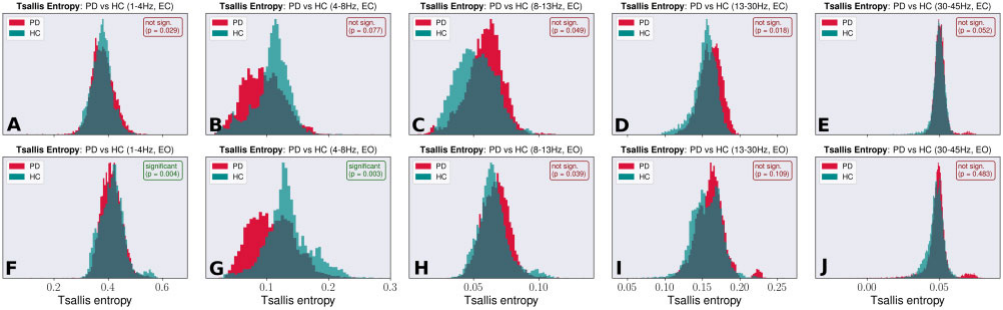
**Tsallis entropy during eyes-closed and eyes-open condition. Normalized Tsallis entropy histograms for the Parkinson’s disease and the HC groups.** (**A**–**E**) the histograms for the δ, θ, α, β and γ-band (left to right) in ‘EC’ condition. (**F**–**J**) The histograms in ‘EO’ condition for the same bands. Given are the uncorrected *P*-values, based on the non-parametric Mann–Whitney *U*-test. Significance threshold is corrected after Holm–Sidak, a step-down method. The θ-band in ‘EO’ condition shows a significant difference between HCs and the patient group and generally, this band displays the least overlap between both groups.

With 42 Parkinson’s disease patients, each patient’s EEG recorded with 213 electrodes, a total of 8946 distinct entropy values were computed for each given band and condition. For the HC group with 24 participants, a total of 5112 entropy values were obtained per band and condition. For both groups, histograms were plotted. To account for imbalance regarding group size, histograms were normalized to allow for better visualization. The TE histograms for each band and condition are shown in Fig. 1. The θ-band in ‘EO’ condition shows a significant difference between HCs and the patient group (P< 0.006). Moreover, this band displayed the least overlap between the patient and the control group, with the EO condition showing a slightly larger separation than the EC condition. Based on this assessment, and assuming that the same pathological process underlies general Parkinsonian pathology as well as Parkinsonian cognitive decline, further investigations were focussed on the θ-band. What motivated this research is the question of whether the difference in signal complexity of EEG between the Parkinson’s disease and the HC group, quantified with TE, contained information about future cognitive decline.

### Relative band power characterized the Parkinson’s disease and healthy control group in the θ-band

Histograms were also calculated for the relative BP and are shown in Supplementary Fig. 3. Calculations were performed analogously to the calculations of the TE histograms. The same normalization procedure used in case of TE histograms was also applied to the BP histograms. Similar to the histograms of TE, the most prominent distinction between the HC and the Parkinson’s disease groups is observed in the θ-band (P< 0.001). But while TE is reduced in patients with Parkinson’s disease compared to the HC group, the inverse is true in case of relative BP.

### Changes in relative band power correlated with changes in Tsallis entropy

**Figure 2:**
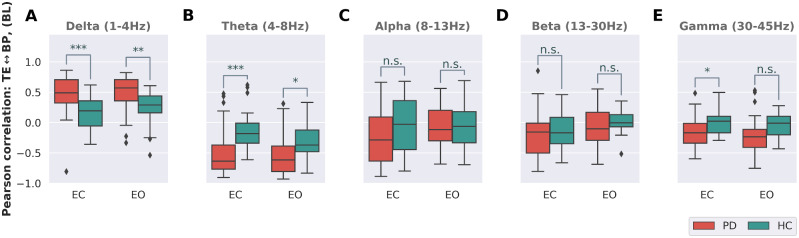
**Correlation between Tsallis entropy and relative band power.** Correlation between Tsallis entropy and relative band power in ‘EC’ and ‘EO’ condition for the patient group and the HCs. Except for the δ-band in **A**, all other bands (**B**–**E**) show a negative correlation between TE and relative band power. This relation is less pronounced for higher frequency bands. Generally, median correlation between signal power and signal complexity of EEG is stronger in the patient group, especially in the range of 1–8 Hz, implying that patients with Parkinson’s disease lose the ability to independently modulate power and complexity of EEG. Significance levels are based on the *t-*test (Holm–Sidak corrected): *P* > 0.005, n. s.; *P ≤ 0.005; **P ≤ 0.001; ***P ≤ 0.0001.

The histograms for TE shown in Fig. 1 and for BP (Supplementary Fig. 3) suggest that a change in BP is strongly correlated to a simultaneous change in TE and vice versa. As a consequence, TE might simply encode the same information as BP did not reveal new information. By assessing the degree of correlation between TE and BP, it is possible to determine whether the complexity of the EEG signal and its relative power are in fact two degrees of freedom that can be regulated independently of each other. Therefore, based on all 213 electrodes, Pearson’s correlation between TE and BP was calculated for each patient as well as the HC group. Figure 2 shows the result for each of the five bands, in both the EO and the EC condition. Generally, the correlation between TE and BP is stronger in the Parkinson’s disease group than HC group, which tends towards a close-to-zero median correlation. Furthermore, correlation is stronger in the lower bands, i.e. δ (P< 0.0001) and θ (P< 0.005), with the θ-band, showing the highest median correlation within the Parkinson’s disease group. Except for the δ-band, which shows a positive correlation between TE and BP, all other bands are either negatively correlated or display a correlation close to zero. On the group level, with respect to correlation, no pronounced differences between EC and EO condition exist.

### In groups, Tsallis entropy differentiates healthy control, mild cognitive impairment and dementia but not cognitive normal

**Figure 3:**
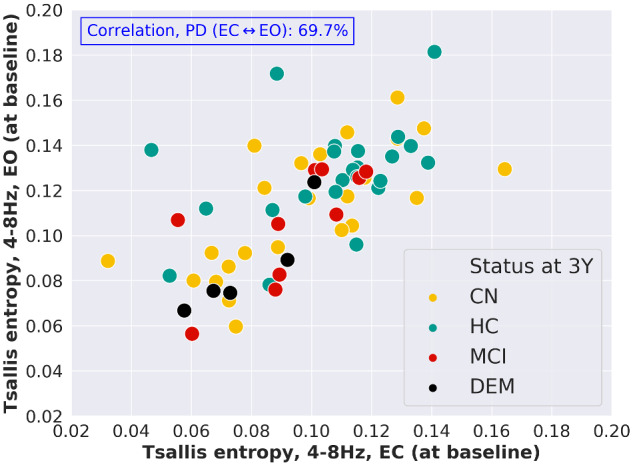
**Tsallis entropy of the θ-band at baseline for the patients suffering from Parkinson’s disease.** Tsallis entropy of the θ-band *at baseline* in EC and EO condition, shown for each subject. The different colours encode cognitive status of each subject at 3-year follow-up. Patients with Parkinson’s disease having developed overt dementia over the course of 3 years are mostly patients with lower baseline TE of the θ-band. For the group of patients with Parkinson’s disease, the relatively low Pearson’s correlation of 69.7% between TE in EC and TE in EO condition, points towards a sensitivity of TE with respect to this condition.

For the participants of the study, the clinical diagnoses at baseline as well as at 3-year follow-up were available. At baseline, the cohort was composed of 24 HC, 31 cognitive normal patients (Parkinson’s disease) and 11 patients suffering from mild cognitive impairment. At 3-year follow-up, only 24 patients were Parkinson’s disease-cognitive normal, 10 patients were suffering from Parkinson’s disease-mild cognitive impairment and 5 patients were diagnosed with Parkinson’s dementia. Figure 3 shows each subject in a two-dimensional plot with the baseline TE level of the θ-band in EC and EO condition on its axes. For patients with Parkinson’s disease, TE estimated in EC condition shows a correlation of 69.7% with TE estimated in EO condition. This is considerably lower than the 83.6% correlation between relative BP in EC and EO condition found for the same patients. The different colours of the individual markers in Fig. 3 indicate the cognitive status at 3-year follow-up, where mostly patients with lower baseline TE have progressed to dementia. This qualitative observation is statistically analysed in the next section.

### Tsallis entropy correlated with 3-year overall cognitive decline in the group of patients with Parkinson’s disease

**Figure 4:**
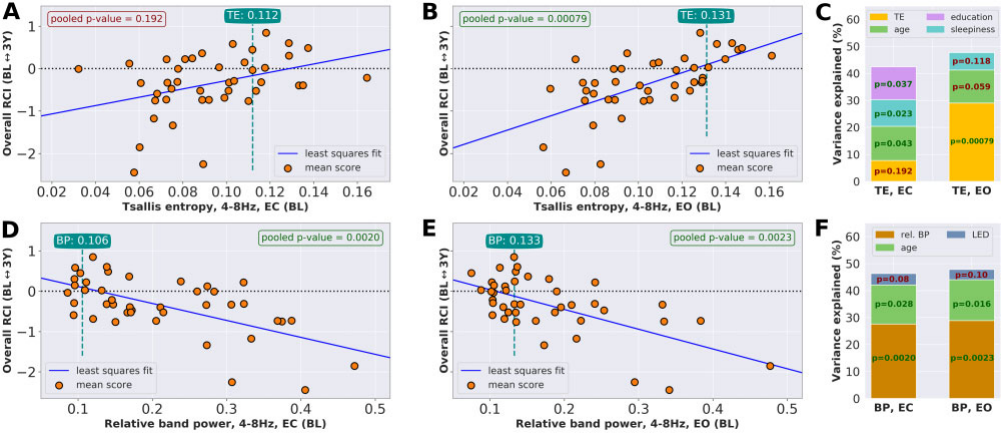
**Relationship between baseline Tsallis entropy of the θ-band and overall cognitive change.** Overall RCI at 3-year follow-up. (**A**–**C**) Tsallis entropy at baseline and overall RCI is correlated significantly only in ‘EO’ condition, where∼30% of the variance is explained by Tsallis entropy. For Tsallis entropy measured in EC condition, the association is not significant. (**D**–**F**) For relative band power, prediction of 3-year cognitive decline is not sensitive to EC/EO condition, where *both* conditions explain∼30% of the variance. *Note*: The median entropy and median relative band power values of the control group in the respective condition are indicated in the green boxes.

Here, cognitive decline is understood as an overall decline of cognitive abilities and is thus quantified based on a combination of RCI scores from multiple cognitive domains. As the RCI is designed to be a standardized score, combining multiple RCI domain scores reduces to an averaging procedure over the individual RCI values. By combining the domain-wise RCI values for ‘attention’, ‘executive function’, ‘visuo-constructive ability’ and ‘working memory’, an overall RCI for 6 months and 3 years after baseline is obtained and evaluated for the 42 patients with Parkinson’s disease, in both EC and EO conditions. The evaluation is based on median TE and median relative BP of the θ-band, where the median was taken over *all* 213 electrodes to obtain *global* median values. Figure 4 shows the overall RCI for each patient 3 years after baseline, dependent either on the patients’ global baseline TE or on the global relative BP. Potentially significant confounders were age, education, sex, disease duration, levodopa equivalent dose (LED) and sleepiness. After performing the stepwise selection on all 20 imputed data sets individually, followed by a majority vote, the confounders to include in the final pooled regression were identified (Table 2). As the overall RCI for the 6-month period was almost negligible, as it is shown in Supplementary Fig. 4, no meaningful regression analysis between either baseline TE or relative BP and the 6-month overall RCI could be performed. The result of the pooled regression analysis for the 3-year overall RCI is summarized in Table 2. Relative θ-BP was significantly correlated with 3-year overall RCI in both EC and EO conditions (P< 0.0020 and P< 0.0023, respectively). For TE measured in EC condition, the correlation with 3-year overall RCI was not significant (P< 0.192), whereas the correlation of TE in EO condition was highly significant (P< 0.00079).

**Table 2:** Pooled linear regression analysis for overall RCI and θ-band TE or relative BP, both for EC and for EO conditions

Time	Condition	*P*Val: TE	Adj. *R*^2^	*P*Val: Age	*P*Val: Sleepiness	*P*Val: LED	*P*Val: Education
θ-band: median Tsallis Entropy ∼ compound RCI							
3 years	EC	0.192	0.42	**0.043**	**0.023**	Not incl.	**0.037**
	EO	**0.00079**	0.48	0.059	0.118	Not incl.	Not incl.
θ-band: median band power ∼ compound RCI							
3 years	EC	**0.0020**	0.46	**0.028**	Not incl.	0.08	Not incl.
	EO	**0.0023**	0.48	**0.016**	Not incl.	0.10	Not incl.

The association of TE in EO condition with overall RCI is more significant than for relative BP in either condition. Significant confounding factor for BP in both EC and EO conditions was age, whereas none of the confounders was significant in case of TE in EO condition. Confounders that were not included into the final pooled regression, following stepwise selection and majority vote, are marked with ‘not incl.’.

In general, the main tendency of higher TE at baseline making a 3-year decline less likely is reversed in case of relative BP of the θ-band, where high values indicated an increased risk of 3-year cognitive decline.

### Tsallis entropy and age explained *>*40% of the variance of the 3-year overall RCI

For all predictors of 3-year overall RCI listed in Table 2, their relative importance was calculated. The results are shown in Fig. 4C and F. For TE in EC condition (Fig. 4A), the association with the 3-year RCI was not significant, which is reflected both by the low explained variance of TE in EC condition as well as by the overall lower adjusted *R*^2^ compared to the other settings listed in Table 2. For TE in EO condition (Fig. 4B) as well as relative BP in both conditions (Fig. 4D and E), age was the second most important contributor to the variance explained. According to Åkerstedt and Gillberg (1990) and Kaida et al. (2006), the correlation between relative θ-BP and self-assessed daytime sleepiness, based on the KSS, is highly significant. Based on the stepwise selection and the subsequent majority vote, both LED and sleepiness were included in the analysis. But neither predictor was below the significance level of 0.05, and only <10% of the variance could be explained by either LED or sleepiness.

### Extreme group behaviour

**Figure 5:**
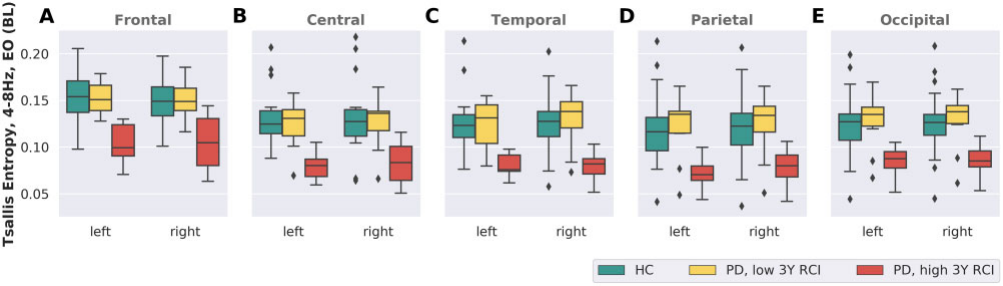
**Median entropy in different brain regions across healthy, cog. stable and cog.** declining participants. Median entropy values are shown for the HCs (green, *n* = 24) as well as for the extreme groups of cognitively stable individuals (yellow, n = 10) and cognitive decliners (red, *n* = 10). The most prominent difference involves a distinctly reduced TE at baseline for patients showing a strong 3-year cognitive decline. The cognitively most stable patients are indistinguishable from HCs based on their regional TE levels. With no apparent regional differences, decreasing TE of the θ-band during the course of Parkinson’s disease is likely a global effect not attributable to a single location.

Global median entropy (θ-band, EO) within the cohort of 42 patients with Parkinson’s disease was in the range of [0.056; 0.161]. For the 10 patients showing the strongest decline (high overall RCI values) over a period of 3 years, the median of the global entropy was in the interval [0.056; 0.105]. The 10 patients with the least decline (lowest overall RCI values) over that same period showed global entropy values in the range of [0.071; 0.161]. Supplementary Table 1 lists the baseline demographic of these two extreme groups. Overall, the median age of the group of strong decliners was 8 years higher than for the group of cognitively stable patients. Furthermore, the strong decliners had a 20% higher median LED. Otherwise, both groups showed similar characteristics. Regional differences in TE of EEG in EO condition between the HC group and the two extreme groups of stable and declining patients are shown in Fig. 5. The extreme group of cognitively stable patients had TE levels in the same range as the HC group *across all regions*. On the other hand, the group of extreme decliners showed lower TE levels, again *across all regions*. Regional differences were thus not present, supporting the hypothesis of decreasing TE in association with Parkinson’s disease being a non-localized effect.

### Stability of Tsallis entorphy estimates and intra-subject variability

**Figure 6:**
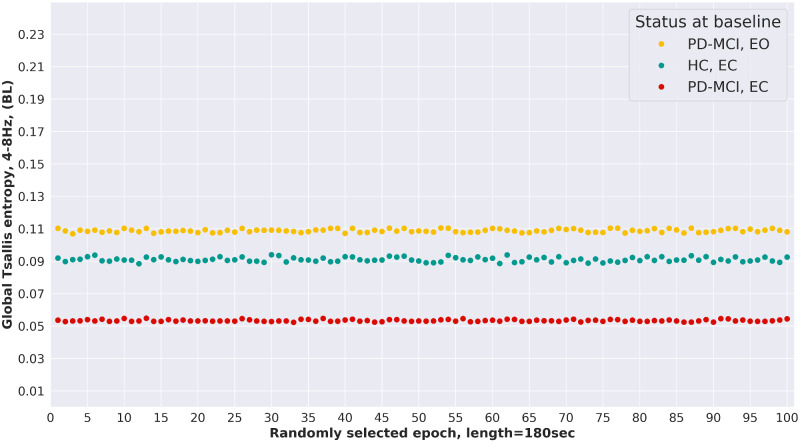
**Stability of Tsallis entropy estimates.** For three study participants, EEG with more than twice the epoch length of 180 s used in this analysis was available. From these recordings, 100 epochs, each with a length of 180 s, were randomly sampled and the global TE of the θ-band was estimated. The results show a very low variance across TE of the different sample epochs, making global TE of the θ-band a robust feature with low intra-subject variability.

From a subset of patients, slightly >360 s of EEG were available after pre-processing, corresponding to approximately double the epoch length of 180 s used in this analysis. For three such patients, global TE of the θ-band was re-calculated a total of 100 times in a random re-sampling setting, where each randomly selected epoch had a length of exactly 180 s. Figure 6 shows the results of the re-sampling procedure. Overall, the global TE estimates show very little variance over randomly selected epochs, which potentially makes global TE, estimated with the procedure proposed by Sneddon (2007), a robust measure of signal complexity of EEG.

## Discussion

θ-band TE measured at baseline in EO condition correlates with cognitive outcome after 3 years in groups of patients with Parkinson’s disease, independently from age, education, sex, disease duration, sleepiness and LED. This effect is highly significant only in EO condition, whereas in EC condition the association with 3-year overall RCI remains non-significant. In accordance with the *a priori* hypothesis, these results indicate that autonomous information is contained in TE. The fact that cognitive decline can be predicted even in a cohort of patients with only a short median disease duration (2.5 years) suggests that sensitivity of TE is already early in the course of the disease.

Daytime sleepiness is an early sign of Parkinson’s disease (Abbott, 2005; Wulff, 2010); moreover, daytime sleepiness in healthy people is associated with an increased θ-synchronization in EO condition (Achermann, 2016). However, clinical measures of sleepiness at baseline are not significantly correlated with cognitive outcome after 3 years (Table 2). This result further supports the conclusion that TE at baseline predicts cognitive decline over middle- and long-time periods independently from any influence that daytime sleepiness might have on short-term outcome or the presence of cognitive ability (Goldman *et al.*, 2014). Moreover, in our cohort of patients with relatively short disease duration and a comparatively long education, Mini-Mental State Examination and low education are not risk factors for cognitive decline, which suggests that EEG has a role to play for prognosis of cognition in Parkinson’s disease.

This idea is supported by related findings. For instance, Klassen (2011) demonstrated that background rhythm frequency and relative power in the θ-band were potential prognostic biomarkers for Parkinson’s disease. The EEG-based biomarker for changes over time in Parkinson’s disease cognitive decline proposed by (Caviness *et al.*, 2015) is a δ-BP (2.5–4 Hz) which correlated best with longitudinal neuropsychological performance changes in Parkinson’s disease. In Zimmermann *et al.* (2015), the authors conclude that global EEG slowing is a marker for overall cognitive impairment in Parkinson’s disease.

Compared to TE, BP is influenced by a variety of unspecific factors, especially skull thickness and distance from the electrical source. The cause of these dependencies lies in the frequency-dependent attenuation by different materials such as bone, galea, skin and other tissue types. Consequently, a power spectrum depends on individual anatomical and physiological features. Under the assumption that frequency-dependent attenuation affects only the amplitude of a signal, but leaves its frequency approximately unchanged, it follows that TE estimates might be less affected by an individual’s anatomy. This is a consequence of the method used to estimate TE (Sneddon, 2007), as it relies only on the ratio between rapid changes (numerator) and slow changes (denominator) of the EEG signal (Supplementary Equation 2). As a result, these entropy estimates might be less sensitive to information encoded in the amplitude of the EEG.

Moreover, as high variability of individual measurements of absolute BP usually precludes their direct inter-individual comparison, it has become standard to compare relative BP, i.e. the power in a frequency band as a percentage of the total power of the signal. While this normalization of BP is the most obvious transformation in order to facilitate inter-individual comparisons, a significant degree of variation will remain due to individual anatomical characteristics. For this reason, Klimesch (1999) suggests an individual frequency adjustment based on individual α-frequencies as an anchor point. In contrast to relative BP, comparing TE on an absolute scale is possible and does not necessarily require any adjustments.

While power-based features have a long history in EEG research, connectivity measures leverage the promising field of network neuroscience to find candidate biomarkers. M/EEG-based connectivity measures as potential biomarkers of Parkinson’s disease progression were investigated by Olde Dubbelink *et al.* (2014), whereas Berendse and Stam (2007) use M/EEG patterns of neural synchrony in patients with Parkinson’s disease to quantify the stage of the disease. While connectivity studies provide a model for detailed understanding of functional interdependency of different cortical areas and its alteration in dementia (Stam, 2014), the determination of connections, and therefore of the graph structure, may be demanding, given the large number of electrodes or sources and the different possible lengths of recording segments (Hardmeier *et al.*, 2014). In contrast, TE might present a shortcut by considering only possible alterations of signal complexity as resulting from an alteration of the underlying graph structure, rather than characterizing the graph in detail.

TE of the EEG quantifies the amount of information contained in this signal and corresponds to the ratio between fast and slow oscillations (Sneddon, 2007). Higher TE of the EEG reflects a higher complexity of the oscillatory brain activity. Oscillations that can be recorded at the surface of the head must come from synchronous discharges of more than 100 million neurons per scalp electrode (Nunez and Srinivasan, 2006), working under the influence of a common pacemaker. Complexity of the recorded signal increases with the number of pacemakers, provided that the ability of the cortex to react to an increasing number of pacemakers is maintained. For normal cognition or consciousness, an optimal range of entropy of the EEG is a requirement (Carhart-Harris *et al.,* 2014). Abnormally increased entropy of brain activity is observed, e.g., in psychedelic states, whereas abnormally decreased entropy is associated with reduced consciousness (Zhang *et al.*, 2001; Carhart-Harris *et al.*, 2014). Decline of cognition may arguably be considered as a first step into loss of consciousness and, therefore, may also be associated with a decline of entropy.

Moreover, a slight reduction of entropy may precede a clinically evident decline of cognition, since at the very first phase of cortical dysfunction, patients recruit all available functional reserves to maintain apparently normal functioning (Peterson *et al.*, 2015). An example for this coping mechanism is the observation that patients with mild cognitive dysfunction ‘stop walking when talking’ (Nieuwhof *et al.*, 2017). TE relates to the complexity of the cortical activity, and therefore, plausibly to the amount or complexity of information that can be processed by the cortex, which in turn is an expression of cognitive capacity. Interestingly, an artificial increase of entropy of oscillatory brain activity produced an improvement in numeracy skills in adults (Cohen Kadosh, 2010, 2013). In encephalopathic and demented patients, partial or absent suppression of the EEG background activity (Berger effect) is a frequent and relatively early finding (Könönen and Partanen, 1993). Therefore, the difference in EEG readings between HC and demented patients is greater when recorded in the EO than in the EC condition. Interestingly, TE shares a similar behaviour and shows significant differences only between Parkinson’s disease patients with and without cognitive decline >3 years when recorded in the EO condition. Moreover, the difference of explained variance by TE measured in the EO condition, as opposed to the EC condition, points to an early deficiency of the ‘orienting response’ (Sokolov, 1963) in the development of Parkinson’s disease dementia, which cannot be detected by BP-based analysis of the EEG or neuropsychological testing before cognitive decline occurs. Loss of capacity to detect unexpected salient changes of environment may be at the base of both the alteration of TE in EO and the beginning of cognitive decline.

The correlation of TE with cognitive outcome is observed when using global EEG, but also when the 10 regions are considered separately, as shown in Fig. 5. Upon visual inspection, the only distinction between the two extreme groups is a shift towards lower TE values across all regions in case of patients with a strong cognitive decline. The difference of the medians between the extreme group of cognitive stable and the group of cognitive declining patients is ∼0.05 across all regions. Comparing the HC group and the extreme group of cognitively stable patients, no differences become apparent: judging only by their TE levels across regions and interpreting TE as a measure of cognitive health, the extreme group of cognitively stable patients with Parkinson’s disease appears *as cognitively healthy* as the HC group. Following Obeso *et al.* (2004), when the capacity of cognitively stable Parkinson’s disease patients to compensate is exhausted, signal complexity in all regions will begin to drop, and eventually reach levels below the median TE levels of HC. The results apply to groups, and may help to define study populations for clinical trials, but cannot be applied in their present form for individual treatment decisions or counselling.

Much effort has gone into the discovery of clinically relevant biomarkers for cognitive decline in Parkinson’s disease (Cozac *et al.*, 2016). While this remains an active field of research, it is very likely that any newly emerging biomarkers will be a composite biomarker, based on more than one physiological or psychological measure. The composite prognostic biomarker for dementia in Parkinson’s disease proposed by Liu *et al.* (2017), for example, is based on age at disease onset, Mini-Mental State Examination, years of education, MDS-UPDRS III, sex, depression and β-glucocerebrosidase mutation status. While psychological testing is affected by test–retest reliability and learning effects, and whereas genetic testing for Parkinson’s disease dementia is still insufficiently validated, there is a practical advantage in having biomarkers derived from signals generally considered to be easily accessible, such as quantitative EEG.

### Significance of the study

Defining cohorts at very high risk of cognitive decline is important in clinical trials for reaching significant results quickly with a relatively low number of patients. While the results of this study contribute to group characterization and, therefore, might help alone, or in combination with other parameters, to select the best groups for clinical trials, they cannot be used in their present form for individual counselling.

### Limitations and strengths

Limitations of this study include unknown reliability of TE. However, for processing the EEG, we used the fully automated TAPEEG (Hatz *et al.,* 2015) because its reliability has been demonstrated. Moreover, only 5 out of the 42 patients with Parkinson’s disease developed overt dementia over the period of observation of 3 years, and the reliable cognitive deterioration was relatively small. Strengths of this study include a carefully matched HC group regarding age, sex and education level, as well as comprehensive neuropsychological testing of the patients with Parkinson’s disease.

## Conclusion

Currently, measures based on EEG for monitoring present cognition in Parkinson’s disease, and predicting its future development, are often derived from BP or connectivity estimates. However, TE is a new measure of signal complexity that seems to be at least as sensitive, and possibly more robust against influence of age, than spectral analysis for predicting cognitive decline in groups of patients, but not individuals. Furthermore, in contrast to BP, TE is sensitive to EC/EO condition, with only the EO condition providing information with respect to cognitive decline over a period of 3 years. This might motivate new neuropsychological testing paradigms specifically designed for the EO condition.

## Supplementary material

Supplementary material is available at *Brain Communications* online.

## Supplementary Material

fcaa207_Supplementary_Data

## Abbreviations

α-band =: 8–13 Hz
β-band =: 13–30 Hz
γ-band =: 30–45 Hz
δ-band =: 1–4 Hz
θ-band =: 4–8 Hz
BP =: band power
EC =: eyes-closed
EO =: eyes-open
HC =: healthy controls
KSS =: Karolinska sleepiness scale
LED =: levodopa equivalent dose
MDS-UPDRS =: MDS-Unified Parkinson’s disease rating scale
RCI =: reliable change index
TE =: Tsallis entropy
