# Supplementary material for: Cognitive decline in Parkinson’s disease is associated with reduced complexity of EEG at baseline
Source: Brain Commun. 2020 Nov 27;2(2):fcaa207. doi: 10.1093/braincomms/fcaa207 (PMC7749793; doi:10.1093/braincomms/fcaa207)
Supplement: fcaa207_Supplementary_Data [file fcaa207_supplementary_data.pdf]

SUPPLEMENT

Cognitive decline in Parkinson's disease is associated with reduced complexity of EEG at baseline

S. M. Keller[1], U. Gschwandtner[2], A. Meyer[2], M. Chaturvedi[1,2], V. Roth[1] & P. Fuhr[2]

1) Dept. of Mathematics and Computer Science, University of Basel
2) Dept. of Neurology, University Hospital Basel

Tsallis entropy calculation of EEG time series

The q-entropy, also known as the Tsallis entropy (TE), was proposed in (Tsallis, 1988) and takes the following form

$$S_q(p_i) = \frac{1 - \sum_{i=1}^{W} p_i^q}{q - 1}$$

Supplementary Equation 1: *For different values of q the occurrence of an event i, associated with the probability of occurrence pi, is weighted differently. The parameter q is a positive real-valued number. For q→1 the well-known Shannon entropy is recovered. The total number of possible events is given by W ∈ N.*

with probabilities p_i and q ∈ R. In the limit q→1, Supplementary Equation 1 recovers the standard Shannon entropy. Due to the parameter q, TE is a so-called parametric entropy, as different values of q result in different weighting schemes of the probabilities p_i. Here, we focus on TE for q=2, which is estimated using the following formula proposed in (Sneddon, 2007):

$$TE_{q=2} = 1 - \frac{\frac{1}{N}\sum s_i^2}{\sigma^2}$$

$$s_i^2 = \frac{1}{n}\sum_{j=1}^{n}\left(x_j - \mu_i\right)^2$$

N: Number of bins

s_i^2: variance within bin i

μ_i: *mean of* bin i

σ^2: variance of entire signal

Supplementary Equation 2: *Estimator of Tsallis entropy for q=2. TE is estimated by calculating the average variance within all bins s_i^2 (fast changes of the EEG signal) divided by the variance of the entire signal (slow changes of the EEG signal) as proposed in (Sneddon, 2007).*

The estimation procedure was as follows. For each electrode, the recorded time series is binned at local extrema, as shown in panel A of Supplementary Figure 1. These bins were usually different in size and contain different amounts of sample points. For example, bin 3 was wider than bin 11 and contained slightly more sample points. Then, for each bin, the within-variance s_i^2 was calculated according to Supplementary Equation 2, where x_j denoted a single sample point and μ_i is the mean of all sample points within bin i. The total number of sample points within each bin is given as n. This operation needed to be repeated for all N numbers of bins. The other quantity needed to estimate the entropy $TE_{q=2}$ was, according to Supplementary Equation 2, the variance σ^2 of the entire signal. This calculation was independent of the previously introduced binning.

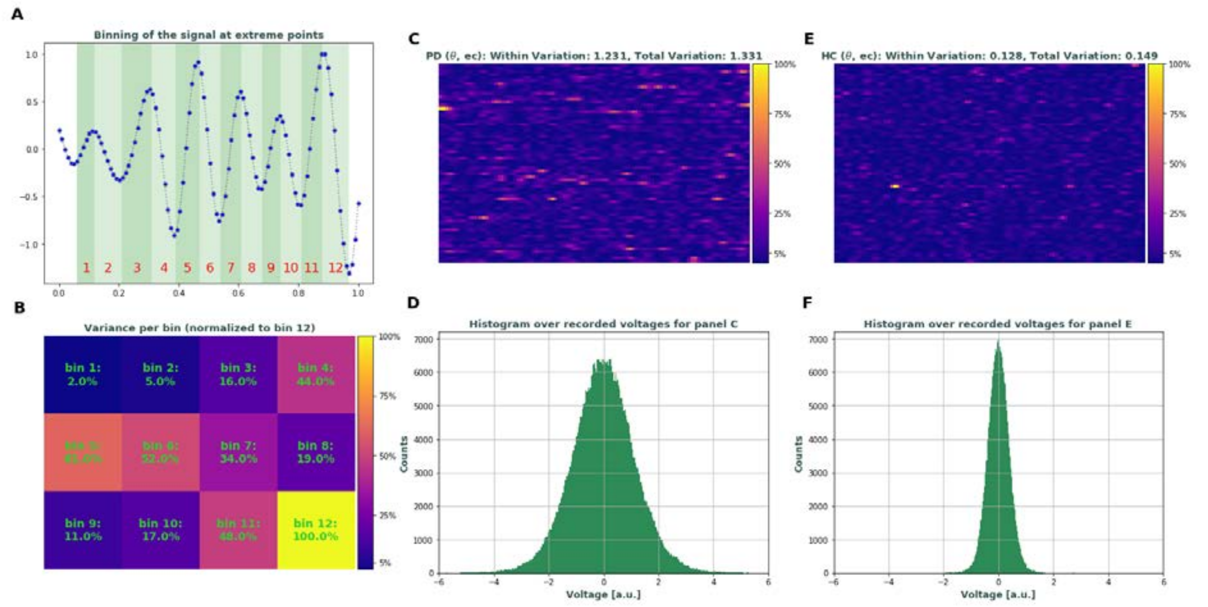

Supplementary Figure 1: *(A) An EEG signal is binned at its extreme values. (B) For each of the twelve bins, the percentage in variance is indicated with respect to bin 12 exhibiting the highest variance, i.e. 100%. (C, E) For 15 minutes of EEG, the signal recorded at a single electrode was binned, and for each bin the variance was calculated. The result is displayed in the same manner as in panel (B). The signal in panel (C) exhibits a lower entropy than the signal in panel (E). (D, F) The histogram over all measured voltages for the signal in panel (C) shows a higher variance than the signal of panel (E). The signal in (C, D) is based on a patient from the PD group, while the signal in (E, F) was recorded on a patient from the healthy control (HC) group.*

Panel B of Supplementary Figure 1 shows the within-variance for each of the 12 bins. The bins were color-coded from blue to yellow for low to high within-variance, and we assigned a variance of 100% to bin 12 as it displayed the highest variance of all 12 bins. In doing so, we then could give a percentage to quantify the amount of variance in each bin relative to the

variance of bin 12. For example, when comparing bin 11 and bin 12, one can see that doubling the variance (bin 11: 48%, bin 12: 100%) does *not* imply the signal in bin 11 to have half the height of the signal in bin 12 (see panel A).

Panels D and F of Supplementary Figure 1 show two histograms based on the same electrode location, but measured on two different subjects. For a given signal, the variance σ^2 of the entire time series is the width of this distribution. With the sum of the within-variance and the variance of the entire signal, an estimate of the TE for q=2 could be computed (Supplementary Equation 2). In panels C and D, the within-variation of two EEG signals with a total duration of 15 minutes is displayed. The histograms in panels D and F are based on the same signals. Both panels C and F were obtained in exactly the same fashion as panel B. The only difference was the much higher number of bins such that single bins were not visually distinguishable anymore.

At that point, this visual depiction of the variances within the different bins already conveyed the impression that there is a quantifiable difference between both EEG recordings, especially since panel C is based on a recording from a patient suffering from PD, while panel E shows a recording based on a subject of the control group. The total variation as well as the within-variation are indicated in both panels C and E. TE can then easily be calculated, e.g. for panel C the entropy content of the signal is 1-1.231/1.331=0.075. A similar calculation reveals that the entropy content for the signal in panel E is nearly twice as high.

In general, as entropies can take on only positive values, the quotient in Supplementary Equation 2 must never be larger than 1 to ensure positivity of the estimated entropy. This in turn implies the sum of the within-variance to be always smaller than the variance of the entire signal. From a purely mathematical perspective, it is possible to create signals that in fact would produce negative entropy estimates based on the estimator in Supplementary Equation 2. Such an occurrence is even probable if the estimator is applied directly to a raw EEG signal, usually containing a multitude of artifacts, especially if these come in the form of severe signal distortions introduced by bad electrode contact with the scalp.

However, an EEG signal pre-processed as described in the main text, is very unlikely to generate negative entropy estimates, which makes this estimator robust for use in practical settings. Assuming an EEG signal mostly free of artifacts, the variance of the entire signal will be larger than the mean variance over all bins. Consequently, the quotient in equation 1 will

be a real positive number in the interval (0,1), which in turn will restrict possible TE estimates to real positive values between 0 and 1.

TE and relative band power provided non-redundant information

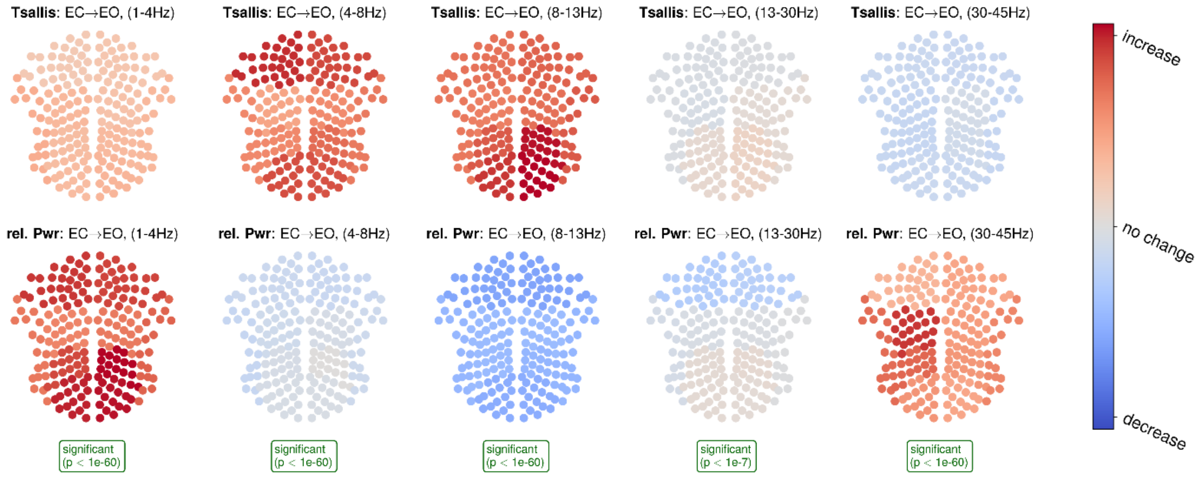

Supplementary Figure 2: *Electrodes were grouped into 10 regions (frontal, temporal, parietal, central and occipital, each left and right). The upper row shows the change in TE of the EO condition relative to the EC condition for the delta, theta, alpha, beta and gamma bands (left to right). Blue (red) stands for a lower (higher) entropy level in EO condition than in EC condition. Color intensity corresponds to the magnitude of change in TE relative to the EC condition. The lower row shows the change in relative band power in the identical setting as for TE. All changes displayed are mean values taken over the 10 individual regions and based solely on the cohort of 24 HC subjects. Significance values indicate that for each band a significantly different effect or reaction of the cortex is captured, depending on whether relative band power or Tsallis entropy is observed. In conclusion, band power and Tsallis entropy capture different aspects of neuro-physiological change and are therefore non-redundant. A multiple testing correction after Holm-Sidak, a step-down method, has been applied. Significance was tested using the Mann-Whitney U-test, a non-parametric rank test.*

Relative band power characterized the PD and HC group in the θ-band

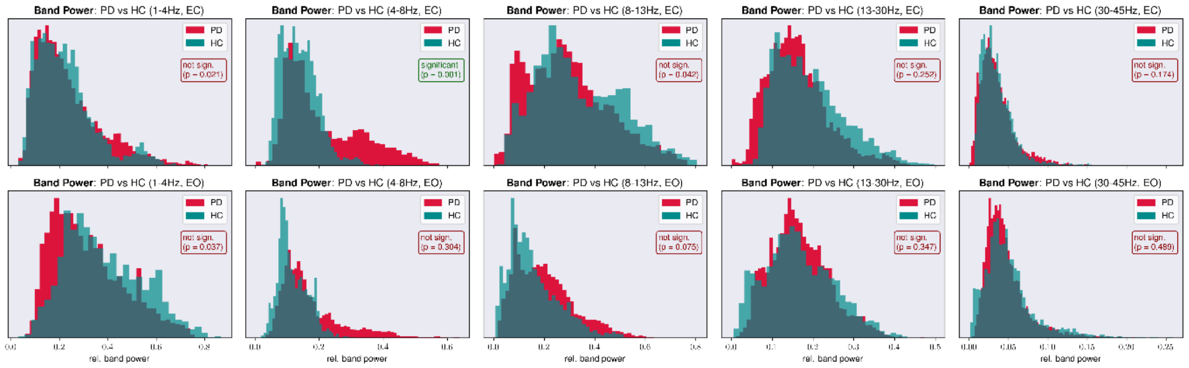

Supplementary Figure 3: *Normalized relative band power histograms for the PD and the HC group. The upper row shows the histograms for the δ, θ, α, β and γ-band (left to right) in EC condition while the lower row shows the histograms in the EO condition for the same bands. A multiple testing correction after Holm-Sidak, a step-down method, has been applied. Significance was tested using the Mann-Whitney U-test. Indicated are the non-corrected p-values while the label 'not sign./significant' was given according to the corrected significance threshold.*

TE and relative BP do not correlate with 6-month overall RCI

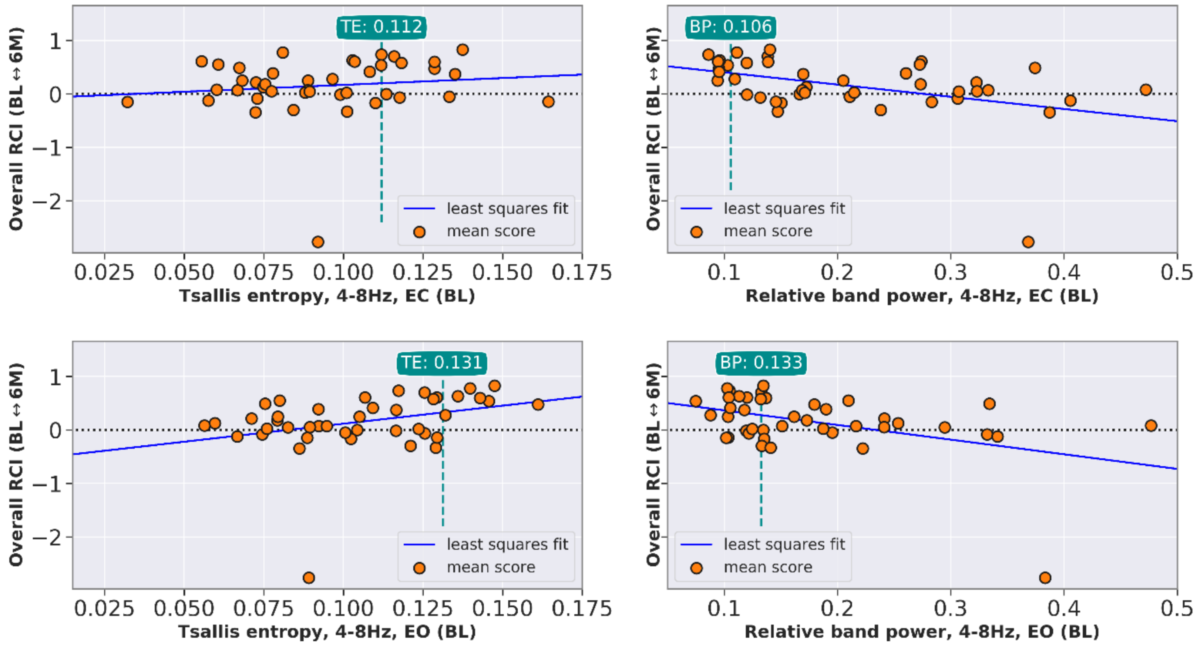

Supplementary Figure 4: *Overall RCI for the 6-month period after baseline. Within this period the cognitive decline is small compared to the statistical noise level. As a consequence, a quantitative analysis is not advisable. Still, the least squares fit shows identical tendencies compared to the 3-year RCI shown in figure 4 of the main text, i.e. positive slope in case of TE, negative slope in case of relative band power.*

Consort scheme

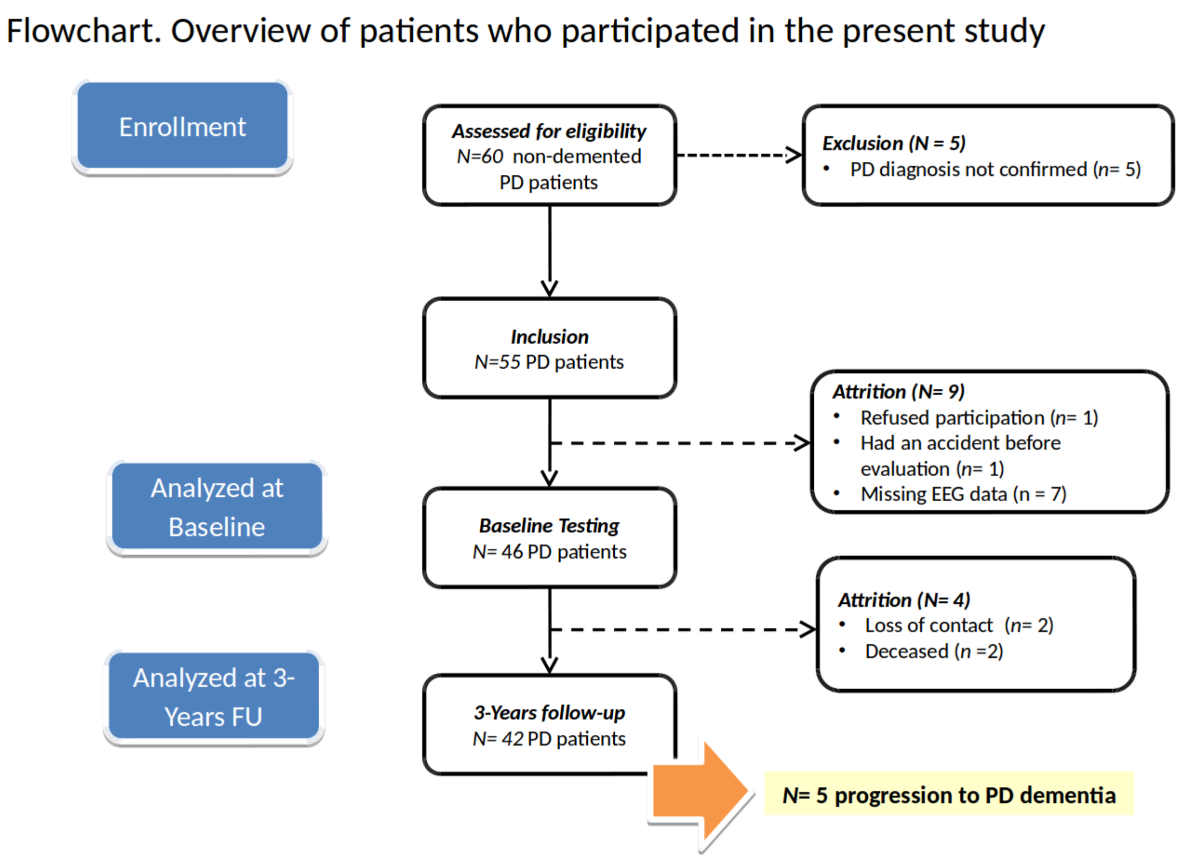

Supplementary Figure 5: *Consort scheme.*

Demographic used in the section on ‘Extreme group behaviour’

	Age	Education	Sex	MMSE	UPRDS-III	LED	Disease Duration	Sleepiness
**PD, low 3Y RCI (10)**	**65.5**	**13**	**6f.**	**29**	**16.5**	**487.5**	**2**	**3**
1st quartile	63.25	12	-	29	7.5	285	0.25	1
3rd quartile	69.75	14	-	29	20.75	560	4.75	3
**PD, high 3Y RCI (10)**	**73.5**	**16**	**3f.**	**28.5**	**15**	**624.5**	**2**	**3**
1st quartile	68.25	13.75	-	28	10	337.5	2	3
3rd quartile	79.25	17.25	-	29	30.25	1335.5	7	6.25

Supplementary Table 1: *Demographic at baseline of the ten cognitively most and least stable patients within the cohort of 42 patients during the 3-year period from baseline. Given are the median values along with the first quartile (25%) and third quartile (75%). Stability is quantified based on patients’ overall RCI value, where a low RCI is indicative of cognitive stability while a high RCI value indicates cognitive decline.*

References

Sneddon R. The Tsallis entropy of natural information. Physica A Statistical and Theoretical Physics 2007; 386: 101–118.

Tsallis C. Possible generalization of Boltzmann-Gibbs statistics. J Stat Phys 1988; 52: 479–487.
